# High-throughput mass spectrometry for screening microbial culture supernatants for aromatic amino acids and selected derivatives

**DOI:** 10.1016/j.btre.2026.e00969

**Published:** 2026-07-08

**Authors:** Amisha Patel, Stefan Stagge, Payam Ghiaci, Leif J. Jönsson

**Affiliations:** a Department of Chemistry, Umeå University, SE-901 87, Umeå, Sweden; b High-throughput Centre, RISE Research Institutes of Sweden AB, SE-892 50, Örnsköldsvik, Sweden

**Keywords:** High-throughput, Mass spectrometry, Aromatic amino acids, Kynurenic acid, Indole-3-acetic acid, *p*-Coumaric acid, Microbial screening

## Abstract

Sustainable production of aromatic amino acids (AAAs) and their derivatives are central to developing renewable routes for aroma- and flavour-related chemicals with myriads of applications in pharmaceutical industry, food and nutrition, cosmetics and personal care, polymer synthesis, and biotechnology. The aim of the present study was to establish and validate a high-throughput mass spectrometry (HT-MS) workflow for rapid screening of AAAs and selected aromatic derivatives, i.e., kynurenic acid, indole-3-acetic acid, and *p*-coumaric acid, using an automated solid-phase extraction system coupled to time-of-flight detection. A method based on fast cycle time, 15 s per sample, and minimal sample handling was elaborated to enable high-throughput screening of large culture collections. Validation using pure standards confirmed excellent linearity (R² ≥ 0.99), accuracy, precision, and on-instrument stability at 4 °C. Although strong matrix effects were observed in microbial culture supernatants, analyte detection remained selective and reproducible, supporting reliable semi-quantitative screening. The results demonstrate that the proposed workflow supports semi-quantitative, comparative analysis of AAAs and their derivatives across large sample sets. Overall, the developed HT-MS method provides a robust and efficient platform for rapid pre-screening of microbial collections accelerating the discovery and optimization of aromatic metabolite production.

## Highlights

• Developed an ultra-fast HT-MS screening platform for aromatic metabolite discovery.
• Automated SPE-TOF enables rapid screening of large strain libraries.
• Robust semi-quantitative profiling in complex culture matrices.
• Provides a scalable tool for bioprocess and strain optimization.

## Graphical abstract

**Figure:**
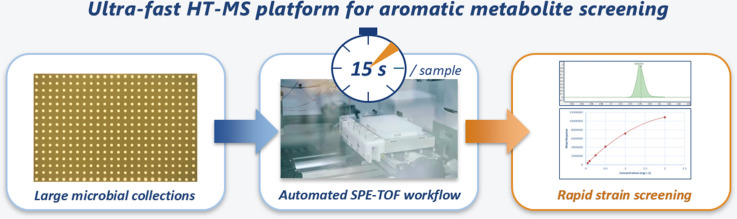

## 1 Introduction

Modern bioprospecting, driven by high-throughput screening [1], is accelerating the sustainable production of bioactive compounds, including aromatic amino acids (AAAs) and their derivatives, which serve as renewable alternatives to fossil-based aromatic chemicals [2]. AAAs and their derivatives are important building blocks in pharmaceutical, food, and industrial biotechnology applications. However, quantifying AAAs and their derivatives in complex biological matrices remains challenging. These metabolites often occur at low concentrations, especially in initial screening experiments. Furthermore, they display considerable structural diversity and coexist with interfering components, such as peptides, sugars, and organic acids in culture supernatants [3].

The shikimate pathway generates aromatic amino acids, l-tryptophan (TRP), l-tyrosine (TYR), and l-phenylalanine (PHE), which serve as precursors for a wide range of industrially relevant metabolites [4]. The functional and economic importance of aromatic metabolites makes rapid screening and comparison of their production across large microbial libraries crucial for identifying strains with superior biosynthetic potential. Such analysis can reveal natural diversity in metabolic fluxes, support strain selection for enhanced bioproduction, and guide metabolic engineering toward high-yielding variants [5]. To advance strain development using systems biotechnology, quantitative data on substrate utilization, product formation, and intracellular metabolite levels are required. These measurements enable closure of carbon balances and identification of metabolic bottlenecks or overflow pathways, providing key insights for rational strain optimization [2].

Conventional analytical methods, including high-performance liquid chromatography (HPLC) and liquid chromatography with tandem mass spectrometry (LC-MS/MS), provide high specificity and sensitivity but are limited by long run times, extensive sample preparation, and low throughput. These constraints make them less suitable for large-scale screening of microbial libraries [6,7]. Automated solid-phase extraction (SPE) platforms offer a faster alternative by enabling rapid sample cleanup and direct coupling to mass spectrometry [8]. Consequently, high-throughput mass spectrometry (HT-MS) has emerged as an efficient approach for rapid, comparative screening of complex metabolite mixtures.

The present study aimed to develop and validate an HT-MS screening methodology integrating a RapidFire automated SPE system with time-of-flight (TOF) MS for rapid analysis of AAAs and their derivatives in yeast culture supernatants. To the best of our knowledge, this is the first reported application of HT-MS for the detection of AAAs and their derivatives, addressing the current need for a fast and reproducible method that requires a minimum of sample preparation, and which is also suitable for large-scale comparative screening. The proposed workflow focuses on analytical throughput, simplicity, and reproducibility, providing a foundation for future high-throughput screening of not just yeast but also varieties of microbial libraries targeting aromatic metabolite production.

## 2 Materials and methods

### 2.1 Reagents and chemicals

Reference standards of TRP, PHE, kynurenic acid (KYNA), *p*-coumaric acid (PCA), indole-3-acetic acid (IAA), and phenylpyruvic acid (PPA) were purchased from Sigma-Aldrich. TYR was obtained from Thermo Fisher Scientific. LC-MS grade methanol, acetonitrile, formic acid, ultrapure ethanol, and ammonium hydroxide were purchased from VWR International. Ultrapure water (18.2 MΩ cm) was obtained from a Sartorius Arium water purification system.

### 2.2 Preparation of standards

Stock solutions (100 mg l^-1^) of TRP and PHE were prepared in ultrapure water. TYR was initially dissolved in 0.1 M hydrochloric acid and then diluted with ultrapure water to obtain a 100 mg l^-1^ stock solution. IAA and PCA were first dissolved in ethanol and subsequently diluted with ultrapure water to yield 100 mg l^-1^ stock solutions. KYNA was dissolved in 50% (v/v) methanol containing 0.5% (v/v) ammonium hydroxide and similarly formulated into a 100 mg l^-1^ stock solution. All stock solutions were stored at −80 °C until use. From these stock solutions, calibration standards were prepared by serial dilution in ultrapure water according to the International Council for Harmonisation (ICH) guidelines for bioanalytical method validation [9]. The prepared standards were subsequently filter-sterilized using 0.2 µm polyethersulfone (PES) filter plates (Agilent 203940–100) by centrifugation at 3000 × *g* for 3 min prior to analysis.

### 2.3 Sample preparation

Three wild type yeast isolates from West African environments, provided by University of Nigeria, were cultivated in minimal medium [10] containing 2% (w/v) glucose as the carbon source and 1.27 g l^-1^ ammonium sulphate as the nitrogen source. Based on these concentrations, the cultivation medium corresponded to an approximate carbon-to-nitrogen (C/N) ratio of 30:1 (w/w). The pH was adjusted to 6.2. Cultures were incubated at 30 °C with shaking at 100 rpm for 40 h. After incubation, the cultures were centrifuged to separate the cells, and the resulting supernatants were collected for further processing. Low- and high-quality control (QC) samples were prepared by fortifying the culture supernatants with the respective reference stock solutions, as described in Section 2.5.3. The fortified samples were subsequently filter-sterilized using 0.2 µm PES filter plates by centrifugation at 3000 × *g* for 3 min prior to analysis.

A subset of 96 culture supernatants, corresponding to one 96-well microplate, was analyzed to demonstrate the applicability of the method for high-throughput screening. These samples were derived from the same yeast collection described above and were analyzed under identical conditions without prior selection for metabolite production.

### 2.4 Automated solid-phase extraction and mass spectrometry

An Agilent RapidFire RF400 automated SPE system (Agilent G9532A) was coupled to an Agilent 6230 Time of Flight (TOF) mass spectrometer (MS) (Agilent G6230B) equipped with an electrospray ionization (ESI) source. Instrument control, data acquisition, and processing were performed using Agilent MassHunter Workstation Software version 11.0. Online SPE was carried out using various RapidFire cartridges prior to sample introduction into the MS. The mobile phases consisted of 0.1% (v/v) formic acid in acetonitrile (solvent A) and 0.1% (v/v) formic acid in water (solvent B). Between injections, the RapidFire sipper was rinsed sequentially with aqueous (water) and organic (acetonitrile) solvents using the integrated wash station. A blank injection was performed after each sample to minimize potential carry-over. MS analysis was conducted in either positive or negative ionization mode, depending on the ionization characteristics of each analyte. The optimized MS parameters for individual analytes are presented in Table 1.

**Table 1:** RapidFire SPE and 6230B TOF-MS operating conditions for analysis of aromatic amino acids and derived metabolites.

	AAAs	IAA	KYNA, PCA & PPA
RapidFire conditions			
Cartridge	HILIC (G9527)	C18 (G9203A)	Phenyl (G9208A)
Cartridge temperature	Room temperature	Room temperature	Room temperature
Injection volume	10 µL	10 µL	10 µL
Pump 1	100% acetonitrile + 0.1% formic acid, flow rate: 1.0 mL min^-1^	100% ultrapure water + 0.1% formic acid, flow rate: 1.0 mL min^-1^	100% ultrapure water + 0.1% formic acid, flow rate: 1.0 mL min^-1^
Pump 2	100% acetonitrile + 0.1% formic acid, flow rate: 1.25 mL min^-1^	10% acetonitrile + 0.1% formic acid, flow rate: 1.25 mL min^-1^	100% ultrapure water + 0.1% formic acid, flow rate: 1.25 mL min^-1^
Pump 3	10% acetonitrile + 0.1% formic acid, flow rate: 0.5 mL min^-1^	80% acetonitrile + 0.1% formic acid, flow rate: 0.5 mL min^-1^	70% acetonitrile + 0.1% formic acid, flow rate: 0.5 mL min^-1^
State 1, Aspirate	600 ms	600 ms	600 ms
State 2, Load/wash	5000 ms	6000 ms	5000 ms
State 3, Extra wash	0 ms	0 ms	0 ms
State 4, Elute	6000 ms	5000 ms	6000 ms
State 5, Reequilibrate	500 ms	1000 ms	500 ms
6230B TOF-MS conditions			
Ion polarity	Dual AJS Positive	Dual AJS Positive	Dual AJS Negative
Gas temperature	180 °C	200 °C	325 °C
Drying gas flow	11 L min^-1^	11 L min^-1^	10 L min^-1^
Nebulizer gas	45 psi	45 psi	35 psi
Sheath gas temperature	350 °C	350 °C	350 °C
Sheath gas flow	12 L min^-1^	12 L min^-1^	11 L min^-1^
Capillary voltage	2500 V	3000 V	3500 V
Nozzle voltage	0 V	0 V	500 V
Fragmentor	120 V	120 V	120 V
Skimmer	65 V	65 V	65 V
Oct 1 RF Vpp	750 V	750 V	750 V
Mass range	50–1000 *m/z*	50–500 *m/z*	100–500 *m/z*
Acquisition rate	4 spectra s^-1^	4 spectra s^-1^	4 spectra s^-1^

### 2.5 Method validation

The developed HT-MS method was validated for specificity, limit of detection (LOD), limit of quantification (LOQ), linearity, accuracy, precision, carry-over, matrix effect, and stability in accordance with the ICH guidelines for bioanalytical method validation [9]. Each validation parameter was evaluated as described in the following sections.

#### 2.5.1 Specificity

Specificity was assessed by analyzing yeast culture supernatants spiked with reference standards to evaluate potential interference at the characteristic *m/z* values and accurate mass windows of the target analytes.

#### 2.5.2 LOD, LOQ and linearity

Stock solutions of each reference standard were diluted to prepare working standard solutions at varying concentrations. The LOD and LOQ were defined as the concentrations providing signal-to-noise (S/N) ratios of ≥ 3 and ≥ 10, respectively, in accordance with ICH guidelines [11]. Calibration curves were constructed by plotting analyte peak response against concentration and fitted using either linear or quadratic regression models.

#### 2.5.3 Accuracy and precision

Quality control (QC) samples were prepared at four concentration levels: the lower limit of quantification (LLOQ); low QC, three times the LLOQ; mid QC, 50% of the calibration range; and high QC, 75% of the upper limit of quantification (ULOQ). Accuracy was determined by dividing the measured concentration of each analyte by its nominal concentration. Within-run (intra-day) accuracy and precision were evaluated by analyzing five replicates at each QC level within a single analytical run, whereas between-run (inter-day) accuracy and precision were assessed by analyzing three replicates at each QC level over three consecutive days.

#### 2.5.4 Carry-over

Carry-over was assessed by injecting blank samples in triplicate immediately after the calibration standard at the ULOQ. If the response observed in the blank sample was <20% of the analyte response at the LLOQ, the carry-over was considered negligible.

#### 2.5.5 Stability

On-instrument stability of the analytes at low- and high-QC levels was evaluated at 4 °C for 72 h. The assessment was performed under the same analytical conditions as described in Table 1, using the RF400 system integrated with a robotic plate handler maintained at 4 °C during analysis.

#### 2.5.6 Matrix effect

Matrix effects (ME) were evaluated to assess potential ion suppression or enhancement caused by yeast culture supernatants under the applied HT-MS conditions. Calibration curves were prepared in pure solvent, and fortified matrix samples were generated by spiking known concentrations of each analyte into yeast culture supernatants obtained from three different yeast isolates. The assessment was performed at two concentration levels (low- and high-QC) following ICH recommendations. Fortified samples were analyzed in triplicate, and mean peak responses were used for calculation. The matrix effect (ME%) was expressed as the ratio of the analyte response in the matrix to that in the pure solvent, multiplied by 100.

## 3 Results and discussion

The HT-MS method was developed for rapid screening of AAAs (TRP, TYR, and PHE) and their derivatives, including KYNA, IAA, PCA, and PPA. Cartridge selection was a critical parameter influencing analyte recovery and signal intensity. Because the RapidFire platform uses SPE for online sample cleanup rather than chromatographic separation, appropriate cartridge was selected based on the observed retention behavior of different sets of analytes within the analytical system.

Hydrophilic interaction liquid chromatography (HILIC) cartridges were employed for the AAAs (TRP, TYR, and PHE), whose zwitterionic nature under the applied conditions promotes hydrophilic and electrostatic interactions, enabling efficient retention and desorption [6]. In contrast, a C18 reversed-phase cartridge was selected for the IAA, whereas a phenyl cartridge provided optimal retention for KYNA, PCA, and PPA. This behavior can be attributed to differences in aromatic character and interaction preferences under the applied eluent conditions, consistent with previous reports [12,13]. Such polarity-based cartridge selection is consistent with previously reported strategies for rapid SPE-MS workflows, where polarity matching between analyte and stationary phase maximizes extraction efficiency and minimizes matrix interference [14].

Among the aromatic metabolites, PCA and PPA share the same molecular formula (C_9_H_8_O_3_) and identical nominal mass (*m/z* 163.0409 in negative mode), rendering them indistinguishable under the present direct-infusion TOF-MS conditions. Consequently, the detected signal at this mass represents a combined response of isobaric species. For method development, PCA was used as a representative compound. This limitation reflects the non-separative nature of the workflow and should be considered when interpreting data from microbial cultures.

Ionization polarity was optimized for each analyte group based on their functional groups and protonation behavior. The AAAs (TRP, TYR, and PHE) and IAA exhibited higher signal response in positive ESI mode due to their amino and indole functional groups, which readily undergo protonation. In contrast, KYNA and PCA exhibited higher signal response in negative ESI mode, consistent with their acidic nature and tendency to form deprotonated ions.

### 3.1 Specificity

**Fig. 1:**
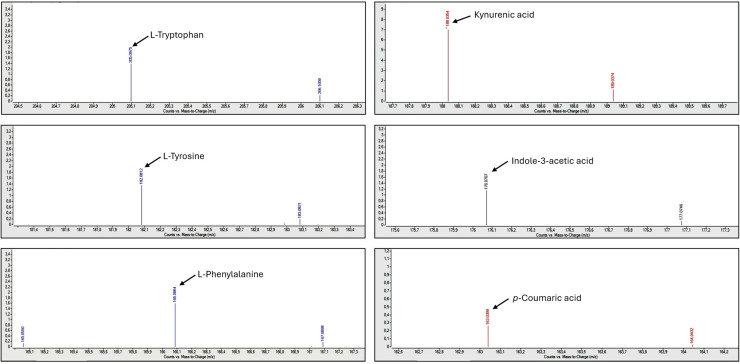
Representative extracted TOF-MS spectra of target analytes in yeast culture supernatants demonstrating specificity in the biological matrix.

The developed HT-MS method demonstrated specificity for all target analytes. Yeast culture supernatants spiked with reference standards were analyzed to assess possible interference at the characteristic *m/z* values and accurate-mass windows of each compound. No background ions or overlapping signals were detected within the predefined mass tolerance (± 10 ppm), confirming that the method can selectively identify AAAs and their derivatives in spiked culture supernatants without chromatographic separation (Fig. 1). The high-resolution TOF analyzer thus provided sufficient mass accuracy and selectivity to differentiate the target analytes from co-eluting matrix components, except for the isobaric pair PCA and PPA, which could not be resolved under the current conditions.

### 3.2 LOD, LOQ, and linearity

**Fig. 2:**
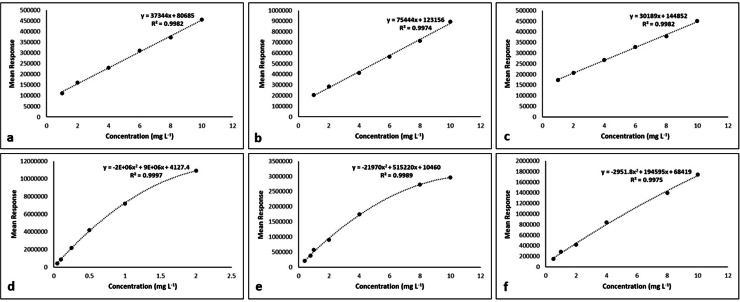
Calibration curves illustrating the working concentration ranges and regression behavior of the analyzed compounds: (a) L-tryptophan, (b) L-tyrosine, (c) L-phenylalanine, (d) kynurenic acid, (e) indole-3-acetic acid, and (f) *p*-coumaric acid.

All analytes showed highly satisfactory calibration performance with coefficients of determination (R^2^) ≥ 0.99 across their respective working ranges (Fig. 2). A linear regression model provided the best fit for the AAAs (TRP, TYR, and PHE), indicating a proportional relationship between concentration and signal response throughout their evaluated ranges. In contrast, KYNA, PCA, and IAA exhibited a non-linear relationship for signal response in the calibration plots at the upper concentration points of their respective calibration ranges, which was better described by a quadratic regression model. This reflects minor deviations from ideal responses commonly observed in direct-infusion TOF-MS systems at extended concentration ranges. Detailed analysis of the LOD, LOQ and calibration range of each analyte are summarized in Table 2.

**Table 2:** Limit of detection, limit of quantification, and linearity ranges for target analytes.

Compound	LOD (mg l^-1^)	LOQ (mg l^-1^)	Calibration range (mg l^-1^)	R^2^
L-Tryptophan	0.03	1.00	1.00 - 10.00	0.99
L-Tyrosine	0.03	1.00	1.00 - 10.00	0.99
L-Phenylalanine	0.03	1.00	1.00 - 10.00	0.99
Kynurenic acid	0.01	0.05	0.05 - 2.00	0.99
Indole-3-acetic acid	0.10	0.40	0.40 - 10.00	0.99
*p*-Coumaric acid	0.01	0.50	0.50 - 10.00	0.99

### 3.3 Accuracy and precision

The accuracy and precision of the method were evaluated at four QC concentration levels for each analyte, and the results are summarized in Table 3. Within-run accuracy values ranged from 91.5%−113.5%, 98.6%−105.8%, 84.8%−108.5% and 85.0%−119.1% for AAAs, KYNA, IAA and PCA, respectively, while corresponding precision values (RSD %) ranged from 0.7%−9.1%, 1.1%−4.4%, 1.4%−3.2% and 2.7%−5.0%, respectively. Between-run accuracy values varied from 99.6%−109.1%, 92.7%−113.7%, 87.5%−107.1% and 87.6%−107.8% for AAAs, KYNA, IAA and PCA, respectively with corresponding precision values of 1.6%−9.2%, 4.3%−10.7%, 3.2%−9.8% and 2.4%−15.2%. According to ICH guidelines, accuracy at each concentration level should fall within ± 15% of the nominal concentration (± 20% at the LLOQ), and precision (RSD %) should not exceed 15% (20% at the LLOQ). All measured values were within these acceptance criteria, confirming that the developed HT-MS method provides reliable accuracy and precision for screening of AAAs, KYNA, IAA, and PCA.

**Table 3:** Within-run and between-run accuracy and precision data for target analytes.

Compound	Concentration (mg l^-1^)	Within-run		Between-run	
		Accuracy (%)	Precision (RSD, %)	Accuracy (%)	Precision (RSD, %)
L-Tryptophan	1.00	99.1	4.9	102.5	9.1
	3.00	106.3	3.5	103.5	1.6
	5.00	97.4	1.3	100.1	2.5
	7.50	87.5	2.0	99.6	9.2
L-Tyrosine	1.00	112.3	4.9	109.1	7.1
	3.00	106.7	3.0	105.4	2.0
	5.00	107.7	0.8	110.8	2.9
	7.50	94.9	0.7	105.9	8.1
L-Phenylalanine	1.00	105.3	9.1	101.8	7.2
	3.00	113.5	3.2	108.1	3.0
	5.00	100.9	2.6	101.8	4.9
	7.50	91.5	1.1	101.4	8.9
Kynurenic acid	0.05	105.8	2.1	113.7	10.7
	0.15	99.2	4.4	93.9	4.3
	1.00	99.7	1.1	95.1	5.1
	2.00	98.6	2.0	92.7	6.0
Indole-3-acetic acid	0.40	84.8	3.2	87.5	3.2
	1.20	108.5	2.4	107.1	4.2
	5.00	95.7	1.5	98.5	4.5
	7.50	105.8	1.4	95.2	9.8
*p*-Coumaric acid	0.50	119.1	2.7	107.8	15.2
	1.50	101.4	5.0	100.4	4.2
	5.00	96.3	3.2	94.5	3.8
	7.50	85.0	3.3	87.6	2.4

**Note:** Within-run precision was determined from n = 5 replicates; between-run precision from triplicate measurements across three consecutive days.

### 3.4 Carry-over

Carry-over was evaluated by injecting a blank sample immediately after the ULOQ in triplicate. The carry-over responses for TRP, TYR, PHE, KYNA, IAA, and PCA were 2.7%, 4.1%, 1.3%, 4.3%, 1.4%, and 5.7%, respectively, which were below the acceptance limit of 20% of the analyte response at the LLOQ. These results indicate that carry-over was negligible under the developed HT-MS conditions.

### 3.5 Stability

The RapidFire plate handler accommodates up to 64 microplates (96-well format) maintained at 4 °C. With an approximate cycle time of 15 s per sample including intermediate washing steps, the total analytical sequence may extend up to 24–72 h. Therefore, on-instrument stability of QC samples was evaluated at 4 °C at 0, 24, 48, and 72 h for the low- and high-QC levels of each analyte. All analytes remained stable over the tested period, with precision (RSD, %) ranging from 0.2%−10.1%, 0.2%−1.5%, 0.3%−5.0%, and 1.1%−4.1% for AAAs, KYNA, IAA and PCA, respectively (Table 4). Corresponding accuracy values ranged from 85.4%−112.8%, 89.7%−97.5%, 86.4%−113.8%, and 85.2%−100.2%. Accuracy at all QC levels was within ± 15% of the nominal concentration and precision was ≤ 15% which are according to ICH M10 acceptance criteria for on-instrument stability.

**Table 4:** Stability of target analytes under instrument operational conditions.

Stability at 4 °C					
Concentration Level		**Low QC**		**High QC**	
Compound	**Time interval (h)**	**Accuracy (%)**	**Precision (RSD, %)**	**Accuracy (%)**	**Precision (RSD, %)**
L-Tryptophan	0	109.0	2.5	86.0	1.0
	24	100.3	5.2	87.3	1.3
	48	104.6	0.4	105.1	3.6
	72	96.5	0.4	105.9	0.9
L-Tyrosine	0	109.2	1.9	94.3	0.2
	24	110.9	2.0	100.1	1.8
	48	109.5	0.8	113.1	2.4
	72	105.5	2.6	111.8	6.8
L-Phenylalanine	0	112.8	2.3	90.9	0.6
	24	101.2	10.1	85.4	3.0
	48	109.0	0.2	104.8	5.4
	72	102.0	1.5	108.1	3.7
Kynurenic acid	0	89.9	1.5	97.5	0.5
	24	89.7	0.4	96.6	0.2
	48	93.4	1.5	93.8	1.5
	72	91.5	0.9	95.7	0.3
Indole-3-acetic acid	0	112.7	1.5	104.1	0.3
	24	113.8	1.6	86.9	1.4
	48	113.8	3.6	102.4	1.5
	72	102.4	2.9	86.4	5.0
*p*-Coumaric acid	0	98.8	1.8	100.2	2.6
	24	96.3	1.1	86.3	2.8
	48	92.7	2.3	96.0	2.8
	72	85.2	4.1	85.5	1.6

**Note:** Stability data represent the mean of triplicate measurements (n = 3) at each time point.

### 3.6 Matrix effect

The results of matrix effects varied widely among analytes and are summarized in Table 5. The three AAAs showed moderate signal enhancement or suppression (ME between 84.5%−130.8%) with acceptable repeatability (RSD ≤ 15%). However, the derived metabolites KYNA, IAA and PCA exhibited more pronounced matrix effects (ME between 6.8–272.3%) and higher variability (RSD > 20%), indicating stronger ionization interference from matrix constituents. These results clearly reflect the chemical complexity of yeast culture supernatants and their pronounced impact on ionization efficiency. Such signal enhancement or suppression is expected in high-throughput analysis of unpurified biological matrices and highlights the intrinsic variability of screening workflows compared with fully validated quantitative assays.

**Table 5:** Matrix effect of target analytes in yeast culture supernatants (n = 3).

Compound	Concentration (mg l^-1^)	Matrix effect (%)	Precision (RSD, %)
L-Tryptophan	3.00	120.0	3.8
	7.50	125.0	6.2
L-Tyrosine	3.00	89.0	2.2
	7.50	84.5	5.5
L-Phenylalanine	3.00	115.0	3.4
	7.50	130.8	11.7
Kynurenic acid	0.15	272.3	68.6
	1.50	184.9	24.4
Indole-3-acetic acid	1.20	7.8	71.3
	7.50	6.8	109.4
*p*-Coumaric acid	1.50	16.5	58.6
	7.50	16.8	65.6

Nonetheless, the developed HT-MS method demonstrated sufficient robustness and reproducibility to support reliable relative comparisons across yeast isolates. In this context, the observed matrix effects do not compromise the interpretability of screening outcomes as the workflow is designed to identify trends and differences in metabolite production rather than provide absolute quantification. Therefore, despite the pronounced matrix influence, the method remains fit-for-purpose as a rapid, semi-quantitative tool for aromatic metabolite profiling in complex culture media. The observed differences in matrix effects among analytes may be attributed to their physicochemical properties and ionization behavior. IAA and PCA, which showed strong signal suppression, are more susceptible to ionization competition from co-existing matrix compounds, leading to reduced signal intensity. In contrast, KYNA exhibited signal enhancement, which may be related to more favourable ionization or reduced competition under the applied conditions.

### 3.7 High-throughput screening of yeast culture supernatants

**Fig. 3:**
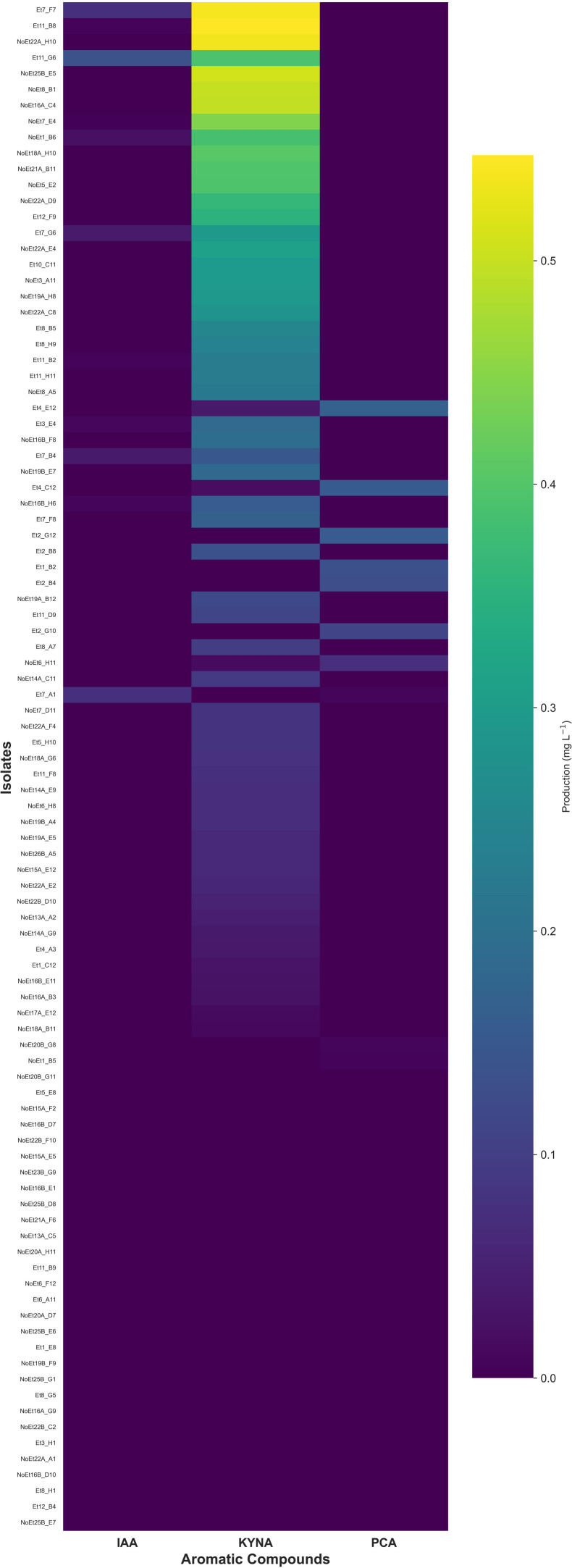
Heatmap visualization of relative HT-MS responses obtained from 96 yeast culture supernatants analyzed as a representative high-throughput screening set. Colour intensity represents relative semi-quantitative signal responses of detected aromatic metabolites (IAA, indole-3-acetic acid; KYNA, kynurenic acid; PCA, *p*-coumaric acid).

To demonstrate the applicability of the developed HT-MS workflow, a set of 96 West African yeast isolates, corresponding to one 96-well microplate was analyzed as a representative high-throughput screening set. The samples were analyzed under identical conditions, minimal medium C/N ratio 30 (w/w), without prior selection for aromatic metabolite production. Under the tested cultivation conditions, aromatic amino acids (TRP, TYR, and PHE) showed no substantial signal responses, whereas derived metabolites (KYNA, IAA, and PCA) exhibited varying relative signal intensities across the analyzed samples (Fig. 3). Relative responses ranged from non-detectable levels to approximately 0.14 mg l^-1^ for IAA, 0.55 mg l^-1^ for KYNA, and 0.17 mg l^-1^ for PCA across the analyzed isolates. The heatmap visualization revealed clear heterogeneity in relative metabolite responses across the analyzed culture supernatants, demonstrating the suitability of the workflow for comparative semi-quantitative screening and relative ranking of microbial culture-derived samples.

Based on an approximate analytical cycle time of 15 s per sample, analysis of a full 96-well plate was completed in ∼24 min. This highlights the potential of the method for large-scale microbial screening workflows.

## 4 Conclusion

A high-throughput mass spectrometry (HT-MS) workflow was developed and validated for the rapid screening of aromatic amino acids and their derivatives (kynurenic acid, indole-3-acetic acid, and *p*-coumaric acid) using an automated SPE TOF-MS platform. The method combines short analytical cycles (∼15 s per sample) with minimal sample handling, offering a practical balance between throughput and analytical robustness. Despite pronounced matrix effects inherent to culture-derived samples, the workflow remains effective for comparative, semi-quantitative analyzes aimed at screening of large sample collections. Validation demonstrated satisfactory precision, accuracy, and stability across a wide dynamic range. Overall, the approach provides a reliable and efficient platform for pre-screening yeasts, and potentially other microorganisms, under multiple experimental conditions prior to detailed targeted LC-MS analysis. The current results demonstrate the performance of the method using reference standards and spiked samples and indicate that it is useful for high-throughput screening of microbial cultures. The inability to resolve certain isobaric compounds, such as PCA and PPA, is an inherent limitation of the method that should be considered in data interpretation.

## Funding

Financial support provided by Kempestiftelserna, Bio4Energy (www.bio4energy.se), and Northfood: Plant Science for Change, a prioritised research area at Umeå University, is gratefully acknowledged.

## CRediT authorship contribution statement

**Amisha Patel:** Writing – review & editing, Writing – original draft, Validation, Methodology, Formal analysis, Data curation. **Stefan Stagge:** Writing – review & editing, Validation, Methodology, Investigation. **Payam Ghiaci:** Writing – review & editing, Supervision, Methodology, Funding acquisition, Conceptualization. **Leif J. Jönsson:** Writing – review & editing, Supervision, Methodology, Funding acquisition, Conceptualization.

## Declaration of competing interest

The authors declare that they have no competing financial interests or personal relationships that may have influenced the work reported in this study.

## Data Availability

Data will be made available on request.
